# Recent advances and perspectives of MicroNeedles for biomedical applications

**DOI:** 10.1007/s12551-025-01317-7

**Published:** 2025-04-24

**Authors:** Renata Faria Maia, Pedro Machado, Raquel O. Rodrigues, Vera Faustino, Helmut Schütte, Stefan Gassmann, Rui A. Lima, Graça Minas

**Affiliations:** 1 Department of Biomaterials & Biomedical Technology, University Medical Center Groningen, University of Groningen, AB Groningen, The Netherlands; 2 Mechanical Engineering Department, MEtRICs, University of Minho, Campus de Azurém, 4800 - 058 Guimarães, Portugal; 3 Center for MicroElectromechanical Systems (CMEMS-UMinho), University of Minho, Campus de Azurém, 4800 - 058 Guimarães, Portugal; 4 Braga/Guimarães, Portugal; 5 Dep. of Engineering, Jade University of Applied Science, 26389 Wilhelmshaven, Germany; 6 CEFT, Faculdade de Engenharia da Universidade do Porto (FEUP), R. Dr. Roberto Frias, 4200 - 465 Porto, Portugal; 7 ALiCE, Associate Laboratory in Chemical Engineering, Faculty of Engineering, University of Porto, Rua Dr. Roberto Frias, 4200 - 465 Porto, Portugal

**Keywords:** MicroNeedles, Drug delivery, Microfabrication, Microfluidic Devices, Fluid Extraction

## Abstract

Microneedles (MN) technology has emerged as a transformative tool within the biomedical field, offering innovative solutions to challenges in drug delivery, diagnostics, and therapeutic applications. This review article provides an in-depth exploration of the diverse perspectives and applications of MNs, shedding light on their pivotal role in shaping the future of biomedical research and clinical practice. It begins by elucidating the fundamental principles of MNs: design, fabrication techniques, and materials, highlighting their capacity for minimally invasive access to the skin and underlying tissues. These attributes have driven advancements in transdermal drug delivery, facilitating precise and controlled administration of therapeutics, vaccines, and biologics, thus improving patient compliance and treatment outcomes. Furthermore, this review investigates the growing range of applications for MNs, including biomarker extraction, interstitial fluid (ISF) analysis, and continuous glucose monitoring. MNs enable real-time and minimally invasive monitoring of biochemical markers and have the potential to revolutionize disease diagnostics, personalized medicine, and wellness monitoring. Their compatibility with microfluidic systems further enhances their potential for point-of-care testing. This review serves as a comprehensive guide, highlighting the breadth of opportunities and challenges in leveraging MNs to improve healthcare outcomes and emphasizing the need for continued research and development in this dynamic field.

## Introduction

Microneedles (MNs) are emerging as a promising tool in the biomedical field, offering a minimally invasive and effective approach to a variety of applications, including drug delivery, cell transfection, and biosensing. MN technology involves the miniaturization of a single needle or an array of needles to micrometer-scale dimensions, allowing for targeted delivery of therapeutics (Meng et al. 2020a), and biomolecules such as proteins (Angkawinitwong et al. 2020), RNA (Ribonucleic acid), DNA (Deoxyribonucleic acid) (Chiappini et al. 2015) into cells, with temporal and spatial precision (Zhuang et al. 2020).

Dimensions of MN may vary depending on the application. However, the most common dimensions found in the literature have height ranges between 100 to 1500 µm, with a base width of 50 to 250 µm and a tip diameter of 1 to 30µm (Waghule et al. 2019). The shape of MNs can also vary including triangular, cylindrical, and pentagonal (Chang et al. 2020). These dimensions play a crucial role in determining the performance and effectiveness of MN systems.

MN design parameters, including shape, number of needles per array, height, aspect ratio (ratio of base to height), material, and thickness of the backing block, significantly impact the volume that can be loaded and administered by the patch. The volume, in turn, influences the choice of MN that best suits the desired outcome (Sonetha et al. 2022). Based on the design and drug delivery strategies, MNs can be classified into two main categories: traditional MN and emerged MN. Traditional MN include solid, coated, and hollow MN, while emerged MN include dissolving and hydrogel-forming MN. Each type of MN offers unique advantages and applications based on the specific needs of the study or of the treatment (Meng et al. 2020b).

MNs represent a departure from traditional drug delivery methods, overcoming challenges such as invasive injections and the limitations of oral administration (Aldawood 2021). By harnessing the power of micro-scale engineering, these tiny structures pave the way for efficient and targeted delivery of pharmaceutical agents (Meng et al. 2020a). MNs are rapidly emerging as a promising drug delivery tool due to their inherent advantages over the conventional drug delivery technologies, which includes their minimally invasive nature. By penetrating the outermost layers of the skin, MNs bypass pain receptors, making the process painless (Kusama et al. 2021). MNs also enhance drug bioavailability by direct delivering drugs into the bloodstream (Hamzah et al. 2012), allowing a controlled and sustained release that ensures stable therapeutic levels over extended periods (Pradeep Narayanan and e Raghavan, S. 2017). Moreover, this versatile technology can be used to deliver a broad range of substances – including vaccines, hormones, and large biomolecules – that are often challenging to administer via conventional methods (Chen et al. 2015; Ita 2018a).

Another important biomedical application of MNs is biosensing and fluid analysis. MN-based sensors enable continuous and real-time monitoring of biomarkers in interstitial fluid, offering immediate feedback on health conditions, such as glucose levels (Ullah et al. 2018; Saurer et al. 2010).

This article aims to offer a thorough overview of the diverse materials employed in MN design, ranging from biocompatible polymers to dissolvable compounds, and the intricate fabrication processes that transform these materials into functional MN arrays. The exploration of MN applications extends beyond the domains of conventional drug delivery. From painless vaccinations to continuous monitoring and diagnostics, MNs demonstrate versatility that extends their utility to various medical and healthcare domains (Kusama et al. 2021).

## Types of MNs

**Fig. 1:**
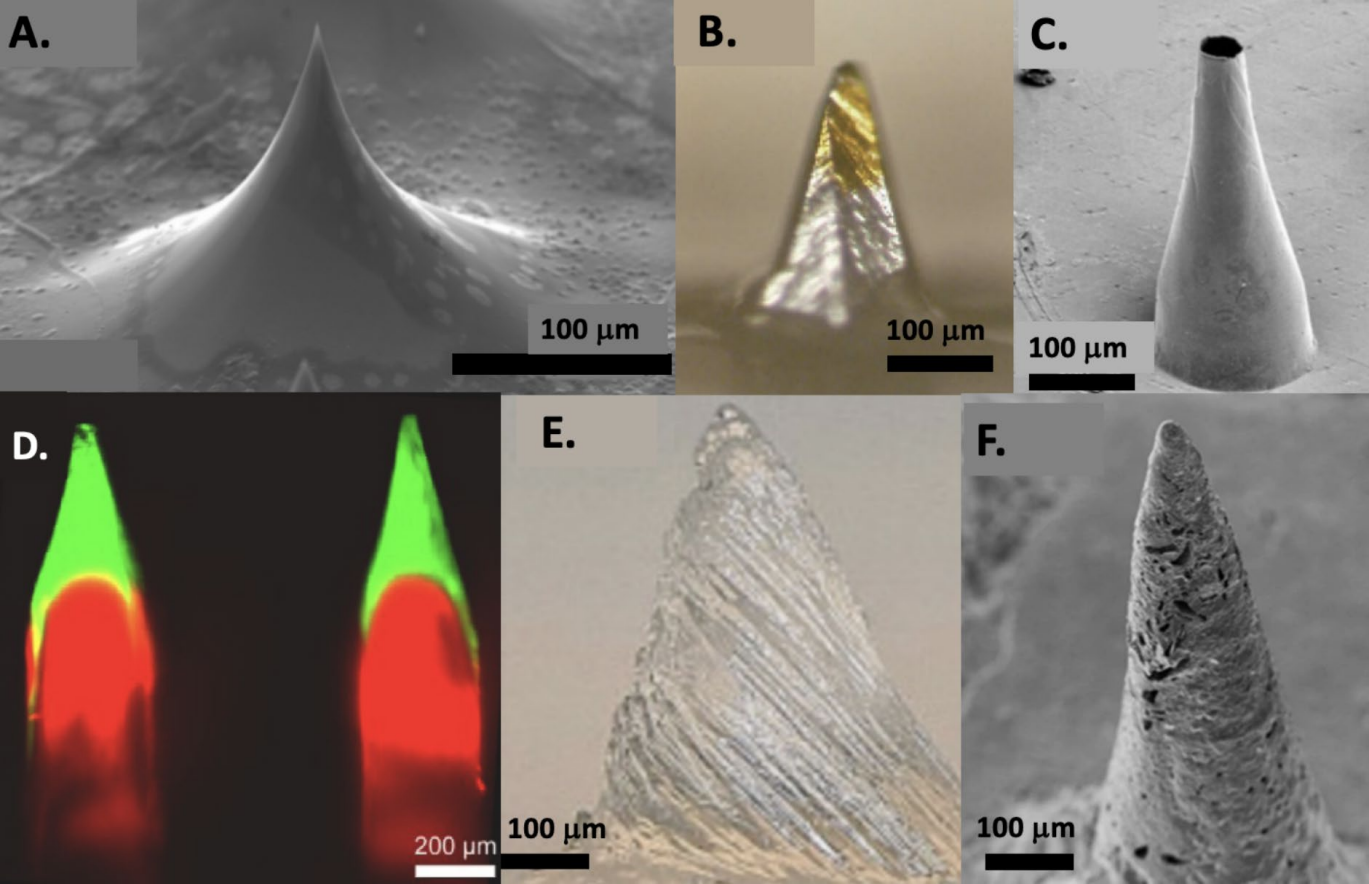
Examples of MNs types. **A.** SMN adapted from Hamzah et al. (2012). **B.** CMN adapted from Chen et al. (2015). **C.** HMN adapted from Davis et al. (2005). **D.** DMN adapted from Yang et al. (2021). **E.** HfMN adapted from Turner et al. (2023). **F.** PMNs adapted from Sadeqi et al. (2022)

**Solid MNs (SMNs)** serve as piercing structures to establish transport pathways or stimulate collagen production in the skin. At times, they also function as electrodes, creating internal channels for drug delivery through residual micropores in the tissue, Fig. 1A (Hamzah et al. 2012). SMNs are typically crafted and developed from metal or other rigid materials (Pradeep Narayanan and e Raghavan, S. 2017).

Traditional, coated MNs (CMNs) are comprised of an SMN base containing drugs or molecules absorbed onto their surfaces in the form of solutions or dispersions, Fig. 1B (Chen et al. 2015). Various techniques, such as spray coating and dip coating, have been developed for coating, but this approach often loses efficiency over time and requires elevated temperatures (Ita 2018a). An additional obstacle arises from the restricted surface area at the disposal (Ullah et al. 2018). A wide range of compounds can be applied as MN coating, including fluorescein, calcein, vitamin B, macromolecules, antigens, DNA, and micron-scale particles (Saurer et al. 2010). In a study by Gill et al. (2007) a patch of 50 MNs was fabricated by assembling ten rows of five MNs. The process involved laser-cutting ten slits into a 1.6-mm thick polyethene medical foam tape to create in-plane rows (Gill and e Prausnitz, M.R. 2007). On the other hand, HyeRin Jeong et al. (2020) focused on enhancing the safety of MN through a coating. They examined uncoated MN of different aspect ratios and widths (135 μm, 118 μm, and 100 μm), all with a common height of 800 μm. The coating, standardized at 200–300 μg (PVA – Poly(vinyl alcohol)) per MN, aimed to minimize post-use skin punctures associated with the MN application (Jeong et al. 2020).

**Hollow MNs (HMNs)** contain an empty cavity within each needle, with a bore at the tip facilitating pressure-driven flow through the needle, as depicted in Fig. 1C (Davis et al. 2005). In contrast to SMNs, HMNs can accommodate larger quantities of drugs or fluids. Various materials, including silicon, metals, ceramics, carbon, and others, along with diverse fabrication methods, are employed to create hollow MN. However, a drawback of HMN is the need of an external pump device or the application of pressure to collect fluid or release drugs from the cavity (Cárcamo-Martínez et al. 2021). HMN where tested to deliver insulin through skin by Davis et al. (2005) in a transdermal insulin delivery in vivo test (Davis et al. 2005). Also, Nicholas et al. (2018), use a polymeric HMN to collect ISF for glucose detection with a monitoring system coupled to a sensor (Nicholas et al. 2018).

**Dissolving MNs (DMNs)** are simple to fabricate and to use as they are built from biodegradable materials, such as polymers and compounds that can incorporate pharmaceutical agents, Fig. 1D (Yang et al. 2021). Upon application and exposure to a watery environment, DMNs seamlessly dissolve (Zhang et al. 2021a) because usually they are made of water-soluble matrix materials, which also avoid biocompatibility issues (Chen et al. 2018). Matrix polymers commonly used to make DMNs are Hyaluronic acid (HA), PVA, Poly(vinyl pyrrolidone) PVP, protein, some carbohydrate compounds, which also allow the incorporation of drugs (Zhang et al. 2021a). The applications of DMN goes from vaccines, to drug release or beauty industry (Zhang et al. 2021a). For example, the use of HA in wrinkle removal, with a matriz of DMN was tested by Hong et al. (Hong, et al. 2017).

**Hydrogel-forming MNs (HfMNs)** represent a distinct subcategory of MNs. To create HfMNs, a bioresponsive system uses swelling polymers that dissolve after MN placement, Fig. 1E (Turner et al. 2023). Upon insertion, the MNs facilitate skin perforation. As the polymers interact with ISF, they expand, opening channels. This approach allows for a controlled release of medication. After device removal, the overall structure of the MNs remains intact (Peng et al. 2021).

Recently, **porous MNs (PMNs)** have emerged as a novel approach, with van der Maaden et al. (2015) pioneering their development (van der Maaden et al. 2015). PMNs feature a substantial proportion of randomly distributed pores, making them well-suited for rapid fluid penetration within their structure, Fig. 1F (Sadeqi et al. 2022; Maia et al. 2024). ISF absorption occurs via capillary forces within the pores, where the interconnected pores enable fluid swelling by the MNs (Liu et al. 2016). However, they are in general fragile due to the extensive porous volume (Liu et al. 2016).

Overall, the exemplified development of MN-assisted and MN-enhanced technologies has led to significant advancements in biomedical applications by overcoming key limitations of traditional MN systems (De Martino et al. 2022). One major innovation involves, as discussed, the incorporation of nanomaterials or compounds, which improves drug loading capacity, enhances stability, and enables controlled-release kinetics. This integration allows for more precise and sustained delivery of therapeutic agents, ultimately increasing treatment efficacy (Dardano et al. 2019). Additionally, MNs can be engineered for site-specific drug delivery, targeting areas such as wounds or tumors (Pei et al. 2025). This localized approach maximizes therapeutic impact where it is needed most, while minimizing systemic exposure and side effects – improving both safety and effectiveness for patients.

MN-enhanced technology also offers the flexibility to be loaded with multiple bioactive compounds, such as antimicrobial agents, growth factors, and stem cells (Li et al. 2025). This multifunctionality supports comprehensive treatment strategies for complex conditions like chronic wounds, where simultaneous action on multiple pathological factors is essential (Huang et al. 2025).

The emergence of these on-demand MNs, fabricated from advanced materials, further addresses common challenges such as limited drug loading and inadequate mechanical strength (Yang et al. 2022a; Yang et al. 2022b). Moreover, the use of biocompatible and biodegradable materials enhances patient safety, reduces the risk of adverse reactions, and eliminates the need for device removal, making MN therapies more patient-friendly and clinically viable (Holicky, et al. 2025).

Moreover, MNs are also being applied as biosensing devices for real-time diagnosis and continuous health monitoring. Particularly, MN-sensors can be integrated into wearable devices, creating a platform for personalized monitoring or chronic disease management (Xiao et al. 2025; Yang et al. 2023).

## Materials and fabrication

The materials used in MN technology can be categorized into groups such as silicon (Si), glass, polymers, metals, or other compounds. Si or Glass faces limitations in terms of cost, fabrication complexity, lengthy processes, and high fragility, thus restricting their widespread use (Hamzah et al. 2012; Mishra et al. 2018; Narayanan and e Raghavan, S. 2019; McGrath et al. 2011; Bolton et al. 2020; Smith et al. 2018; Tang et al. 2018a; Ma et al. 2006; Wilke et al. 2005; Dervisevic et al. 2022). A variety of polymers like poly(methyl methacrylate) (PMMA), Poly(vinyl acetate) (PVa) and poly(styrene) (PS), offer distinct advantages for fabrication such as biodegradability, high biocompatibility, creation of vital hydrogels, excellent mechanical strength, flexibility, and controlled drug release through programmed degradability (Li et al. 2017; Dardano et al. 2021; Barrett et al. 2019; Barrett et al. 2015; Miller et al. 2011; Srivastava et al. 2015; Faraji Rad et al. 2021). Metals, on the other hand, pose some concerns due to the possibility of tissue damage and increased trauma (Mansoor et al. 2013; Ranamukhaarachchi et al. 2016; Kim and e Lee, J.B. 2007). Other interesting materials such as carbohydrate polymers, or inorganic polymers have been extensively used (Mukerjee et al. 2004; Jina et al. 2014; Yu et al. 2009; Roxhed et al. 2007; Bhadale and e Londhe, V.Y. 2021; Liu et al. 2020). Table 1 provides a summarized comparison of the primary materials comprising the MN structures.

**Table 1:** Materials used in MN technologies and their advantages and disadvantages

Materials	Advantages	Disadvantages	Refs
Metal	Stiffness, avoids breakages	-	Mansoor et al. 2013; Yuan et al. 2025; Hsiao et al. 2023)
Ceramic	Chemical resistivity Biocompatible ceramics boast superior mechanical strength and stability in harsh environments, such as elevated temperatures and humidity, compared to many polymers Provide controlled porosity and convenient production handling Offer adjustable porosity and the potential for enhanced percutaneous drug penetration through electrostatic interactions with the ceramic surface	Prone to fracture when subjected to tensile forces	Cai et al. 2015; Cai et al. 2014; Ita 2018b)
Silica Glass	Production flexibility Generating needles of different sizes and shapes	Complex fabrication Not into the market Cost and time-consuming Biocompatibility concerns (grooving likelihood)	Narayanan and e Raghavan, S. 2019; Tang et al. 2018a; Dervisevic et al. 2022)
Carbohydrate polymers (hyaluronic acid, chitin, chitosan, chondroitin sulphate, cellulose, and starch)	Cost-effective and safe for human health (Intrinsically biocompatible, degradable, nontoxic, and sustainable) Innumerable topographies	Not suitable for high temperatures (Degradation above 60 °C) Likelihood of deterioration, degradation, or contamination of these assets during the extraction and advancement processes	Turner et al. 2023; Peng et al. 2021; Li et al. 2025; Alfalasi et al. 2025)
Polymer	Wide range of possible properties Tougher than glass and ceramics Come in various forms and shapes Cost-effective and suitable for mass production and disposable applications	Relatively lower strength	Dardano et al. 2019; Barrett et al. 2015; Wang et al. 2025)

The literature has detailed several MN fabrication methods, including the most common prevalent micromoulding, microfabrication technologies (e.g., lithography, laser cutting/abrasion, etching), and additive manufacturing (injection moulding and 3D printing). Additionally, layer-by-layer assembly is also employed (Economidou, et al. 2021; Yadav et al. 2021). Consequently, microfabrication can be categorized into three primary processes: *deposition* (e.g., chemical vapour deposition, electrodeposition, and casting), *patterning* (e.g., lithography), and *etching* (Lin et al. 2025). However, these methods are limited as they require costly specialized equipment, advanced production facilities, and skilled personnel for large-scale production. To fabricate MNs two approaches can be used: direct fabrication of MNs or fabrication of a mould to produce the MNs (micro moulding). The 3D MN structure with specific height and pitch can be directly fabricated using methods such as photolithography, etching, laser engraving, micromilling, and 3D printing. These techniques are primarily used to create the MN. For the actual molding production, microinjection molding and two-photon polymerization are specifically employed (Yadav et al. 2021).

**Fig. 2:**
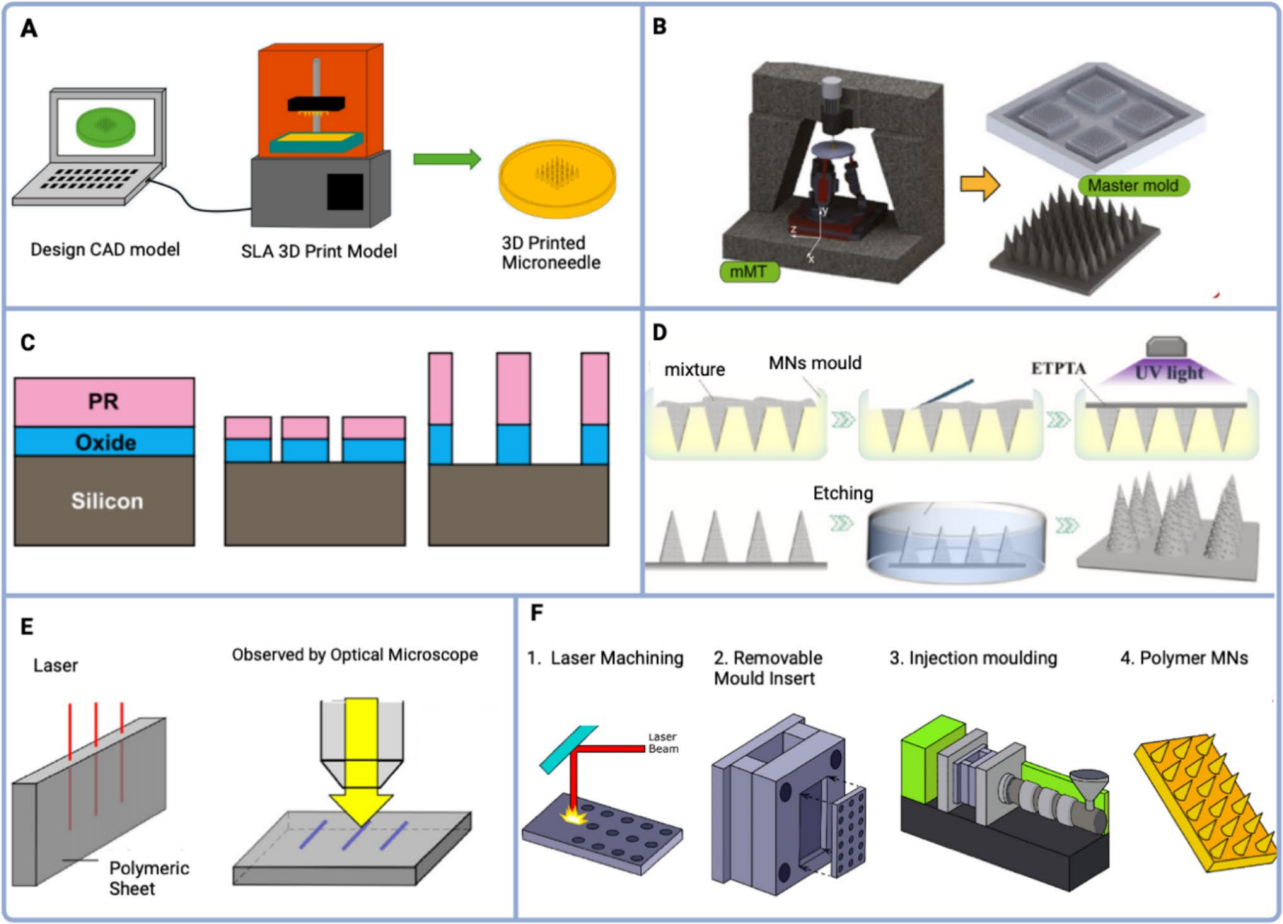
Fabrication methods to produce MNs. **A)** 3D printing adapted from Krieger et al. (2019); **B)** Micromilling adapted from Bediz et al. (2014); **C)** Etching adapted from Tang et al. 2018a; **D)** Micro-moulding adapted from Yi et al. (2021); **E)** Laser ablation adapted from Aoyagi (2007); and **F)** Injection moulding process adapted from Evens et al. (2020)

3D printers commonly used for printing plastic materials include fused deposition modelling (FDM), digital light processing (DLP) and stereolithography (SLA), Fig. 2A (Krieger et al. 2019). SLA offers high-quality and high resolution (ranging from 50 µm down to < 5 µm), building 3D structures layer-by-layer polymerizing photosensitive resin in a UV-light-filled tank, but is slow (time-consuming) and expensive. At the same time, digital light processing is rapid and cost-effective but has limited mechanical properties. SLA printers, with their high-quality surfaces, have opened up possibilities for a wide range of materials, however, most materials used with SLA and DLP are not biocompatible (Venzac et al. 2021; Chan et al. 2015). In addition, the 3D dispensing method allows for a wide range of materials but requires the ones with narrow viscosity (Pere et al. 2018; Singh et al. 2017; Kitson et al. 2012). For example, PolyJet provides fast printing but exhibits weak adhesion between layers (Ligon et al. 2017). Regarding deposition, fused deposition modelling offers high speed and low cost but weak mechanical properties. It can print biocompatible materials such as poly(lactic acid) (PLA) but the resolution is lower than other printing methods (Luzuriaga et al. 2018).

Micromilling can create precise master moulds from materials like PMMA, metal, or ceramic. This method relies on subtractive manufacturing principles, selectively removing material from a workpiece, Fig. 2B (Bediz et al. 2014). The final quality of the micromilled part hinges on various factors, including the condition of the workpiece and process parameters such as tool geometry, spindle speed, feed rate, and depth of cut. Optimizing these parameters can effectively minimize surface roughness. In the realm of research, a range of geometries, such as square pyramidal, obelisk, and tapered obelisk with fillets, have been skillfully fashioned using PMMA templates, employing diverse cutting tools designed like needles. The integration of micromilling with finite element analysis facilitates precise design adjustments (Bediz et al. 2014). Nevertheless, challenges persist in translating intricate moulding processes into high-throughput manufacturing, limiting broader application. Despite these challenges, these two techniques remain robust tools for efficient and standardized MN production (Yadav et al. 2021; Tarbox et al. 2018).

In what concerns subtractive technologies such as Deep Reactive Ion Etching (DRIE), it has emerged as a versatile and precise technique for manufacturing MN with intricate designs and high aspect ratios. Nevertheless, some bottlenecks have been pointed out, since DRIE requires complex and expensive instruments as well as cleanroom facilities with unique treatments and complicated, time-consuming processes to control the operation variables (Jenkins et al. 2012; Kashaninejad, et al. 2021; Coffey et al. 2016). Mostly applied in MN electrode construction, wet chemical etchant studies for MN fabrication have explored different etchant compositions and concentrations to achieve controlled and efficient etching of MN structures, Fig. 2C (Tang et al. 2018a). Researchers have focused on optimizing process parameters such as etching time, temperature, agitation, and drying conditions for achieving desired MN dimensions, aspect ratios, and mechanical properties (Hamzah et al. 2012). It can be created MN with a consistent thickness in one direction, even though it cannot generate tapered or pyramidal MN, neither it can be produced a sharp-tip MN (Lee et al. 2016; Chinnadayyala et al. 2018).

On the other hand, micromolding technology offers high precision and reproducibility due to intricate mold designs, enabling consistent MN manufacturing, alongside mass production, and customization, allowing for efficient scaling and tailored designs. It involves cutting tools to sculpt the mold, pouring the polymeric material into the micro-mold in a liquid or semi-liquid state, and then solidifying it to achieve the desired shape, Fig. 2D (Yi et al. 2021). However, limitations encompass increased production costs and complexity associated with mold fabrication, potentially limiting its feasibility for small-scale applications. Additionally, intricate mold designs might lead to challenges in mold release and maintenance. It takes advantage of polymer materials, and it is the common method used to apply photoresist to substrates (e.g., photolithography). Moreover, to ensure sharp tips and fully filled corners, it is important to utilize techniques like centrifugation or vacuuming during the fabrication process. Researchers have explored various parameters such as mixing ratio, curing conditions, and mould designs to optimize the fabrication process and achieve high-quality MN (Yi et al. 2021).

Two/multiphoton polymerization is a micromolding process which achieves high-resolution quality but is slow in fabrication. Powder-bed-based methods like laser sintering/melting provide high stability and mechanical properties to fabricate metal and polymeric MN, but are slow, expensive, require higher power consumption and result in rough surfaces at high temperatures. Laser ablation, employed as a top-down approach in material processing, has found extensive use in micro-drilling and cutting processes, Fig. 2E (Tarbox et al. 2018).

Conversely, injection moulding shows up as a highly efficient and reproducible technique for mass production, commonly used for thermoplastics MN (like poly(carbonate) (PC), PS, and poly(propylene) (PP)), although not suitable for the fabrication of preloaded sensing materials due to melting during the manufacturing process, Fig. 2F (Evens et al. 2020). Commonly used fabrication processes for solid MN encompass techniques such as casting, injection molding, DRIE, and wet chemical etching (Kashaninejad, et al. 2021). SMN is typically crafted from metallic components or silicon, often employing micromachining methods like SU- 8 photoresist (Jiang and e Lillehoj, P.B. 2020). Regarding HMN and SMN, the fabrication techniques spotted (electrochemical etching and laser ablation) are complex, time-consuming and costly compared to the PMN, requiring expertise and meticulous process control (Mukerjee et al. 2004; Chua et al. 2013). In the realm of mold design, studies have explored diverse approaches, including silicon molds, 3D-printed molds, and micromachined molds, to create MN arrays with precise dimensions and geometries (Au, et al. 2016). Additionally, investigations into surface modifications of the molds have been conducted to facilitate MN release and prevent adhesion during the demolding process. Techniques such as surface coatings, texturing, and the use of release agents have been explored to enhance the mold's performance (Lobita et al. 2023; Martínez-Navarrete et al. 2024).

## Applications

MNs were initially developed as a pain-free alternative to hypodermic needles, given their ability to penetrate the epidermis (Amani et al. 2021). Subsequently, researchers have explored diverse biomedical applications for MNs. One notable application involves MNs serving as a non-invasive tool to interact with the cellular environment, potentially functioning as a direct platform for sensing biological systems. This capability allows for the investigation of various biological systems, spanning from individual cells and biological fluids to tissues and living organisms. Intracellularly, MNs can be employed to detect the electrical activity of excitable networks, as well as to assess the concentration, function, and interaction of biomolecules in situ (Chiappini 2017).

**Fig. 3:**
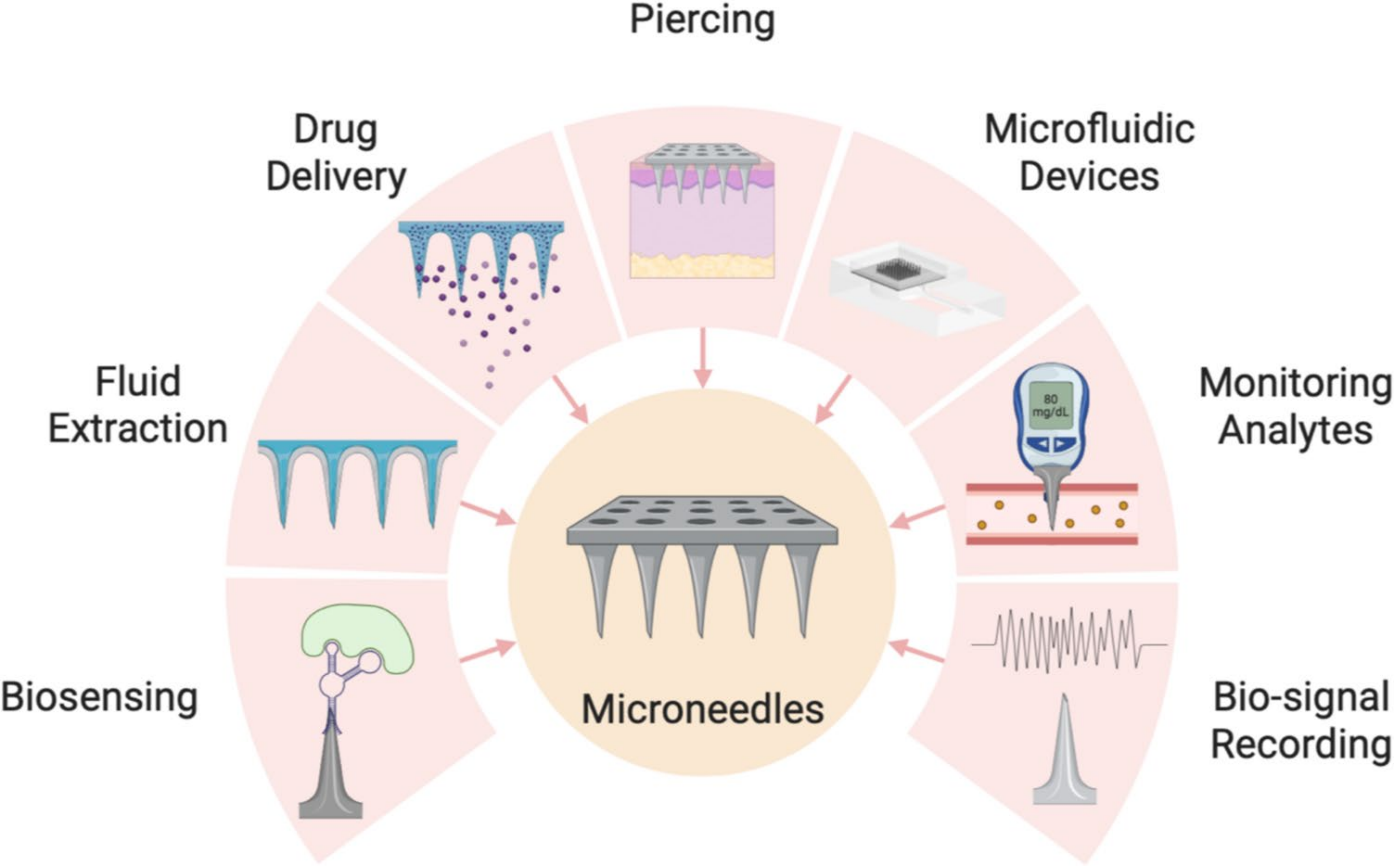
Schematic representation of MN applications in biomedical engineering

Research studies focusing on applications of MN commonly used MNs for drug delivery, transdermal penetration and fluid extraction. They can be integrated into microfluidic devices and also to measure physiological parameters, which mostly involve physical measurements and detecting biomarkers (Ingrole et al. 2021). In the broader branch of biological analysis, MN-based devices can be split into classes: MNs designed for transdermal sampling, capturing biomarkers, detecting or monitoring analytes, drug delivery, and recording biosensing and bio-signal (Fig. 3). Transdermal sampling involves using MN to collect samples from the skin (ISF), whilst biomarker capture refers to the use of MN to capture specific biomarkers of interest. On the other hand, MN sensors are designed to function as sensors for detecting various analytes, alongside bio-signal recording devices, which entails using MN for recording the biological signals (Moreira et al. 2019; Ma et al. 2023).

MN have been used in cosmetics for delivering skincare products or improving the appearance of the skin. As an overview, MN technology has been explored for various applications and therapies, including oligonucleotide delivery (Avcil and e Çelik, A. 2021), vaccine therapy (Gill and e Prausnitz, M.R. 2007; Jenkins et al. 2012; van der Maaden et al. 2018), peptide delivery (Peng et al. 2021; Wang et al. 2021a), hormone delivery (Ameri et al. 2010), cosmetics (Yang et al. 2019), pain therapy (Wang et al. 2021b; Chakraborty and e Tsuchiya, K. 2008), ocular delivery and cancer therapy (Moreira et al. 2019; Hao et al. 2017).

### Piercing and drug delivery

A primary application of MNs is to facilitate the delivery of various substances into the body. This involves employing diverse strategies to administer drugs through micron-sized projections, directing them straight to the region of the epidermis and upper dermis. This allows the drug to enter the systemic circulation directly, bypassing the barrier presented by the stratum corneum layer (Zhang et al. 2021b). They can carry different types of small molecules, biomolecules and antigens (Ingrole et al. 2021). These substances can be delivered through the skin, eyes, and other tissues, providing targeted and precise administration. In summary, this technology is characterized by rapid onset of action, self- administration, enhanced patient compliance, and improved permeability and efficacy. Additionally, MN provide highly precise and reproducible results with minimal inter-subject variability in bioavailability (Avcil and e Çelik, A. 2021).

**Fig. 4:**
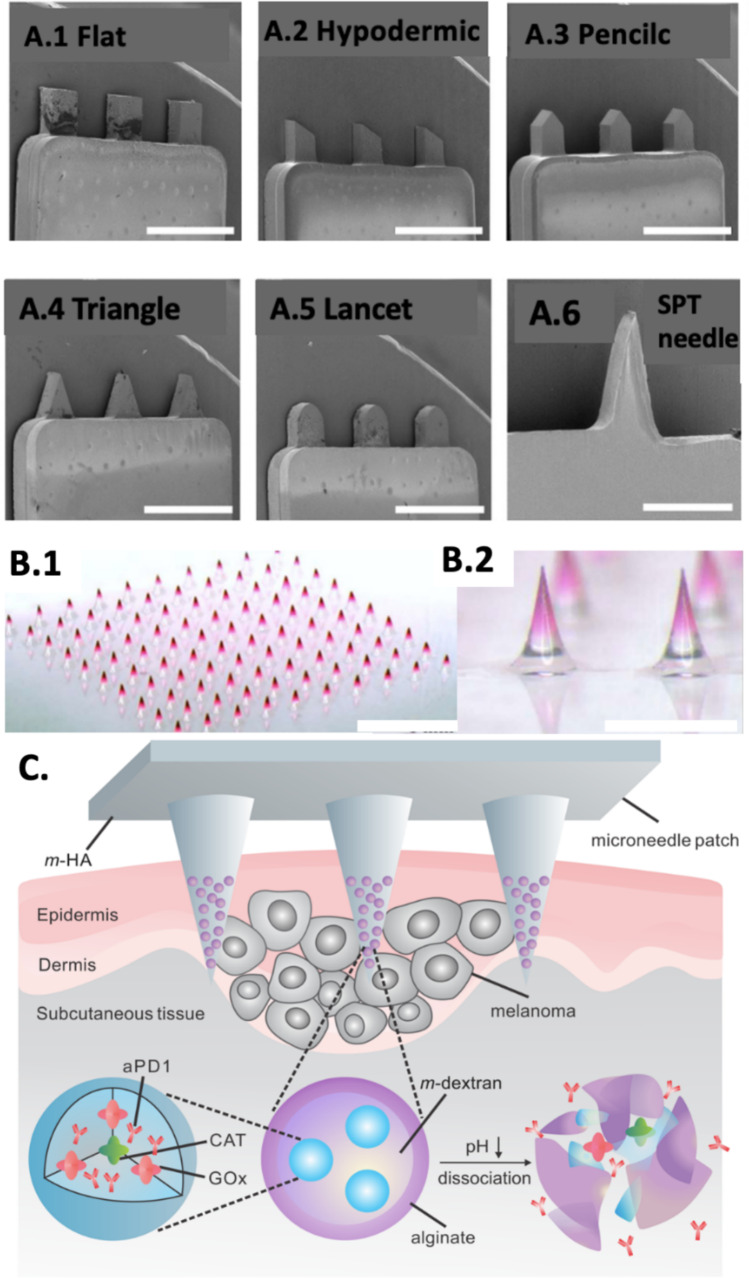
A. SEM images showing the different MN shapes (flat (control) (**A.1**), hypodermic (**A.2**), pencil (**A.3**), triangular (**A.4**), and lancet (**A.5**)) used in this study (L500, W400, T500), along with the skin prick test (SPT) needle for comparison (**A.6**). Scale bars represent 1 mm. Adapted from Bisgaard et al. (2023) **B.** Morphology of the fabricated drug-loaded MNs. (**B.1**) Overall and (**B.2**) enlarged view of Rhodamine B-loaded MNs. Scale bars represent 1 mm. Adapted from Chen et al. (2023) **C.** Schematic of the MN patch-assisted delivery of aPD1 for skin cancer treatment. Schematic of the aPD1 delivered by an MN patch loaded with physiologically self-dissociated NPs with GOx/CAT enzymatic system immobilized inside the NPs by double-emulsion method, the enzyme-mediated conversion of blood glucose to gluconic acid promotes the sustained dissociation of NPs, subsequently leading to the release of aPD1. Adapted from Wang et al. (2016)

In the context of MNs for piercing, it is crucial to delve into factors such as penetration force, depth, and minimizing tissue damage. The penetration force plays a vital role in ensuring that MNs can effectively create a channel within the epidermal region. When determining the depth of penetration, careful consideration should be given to the specific region being targeted. Additionally, efforts should be made to minimize tissue damage, emphasizing the need for a delicate balance between effectiveness and the preservation of surrounding tissues (De Martino et al. 2022). Several studies try to find the best shape, material, and size to pierce the skin. Bisgaard et al. 2023, investigated the influence of MN dimensions and shapes on their penetration force, penetration depth, and tissue damage, Fig. 4A (Bisgaard et al. 2023). The study aimed to evaluate the dermal tissue penetration of in-plane silicon MNs for potential MN-based sensing in the ISF. The MNs with a length of 500 µm were found to be insufficient for reliable detection of skin penetration. Thus, only the long MNs with a length of 1000 µm were considered for further analysis. The MNs with triangular shapes required the lowest force for skin penetration, followed by pencil- shaped and flat MNs. The penetration force was lower for MNs with a width of 200 µm compared to the ones with a width of 400 µm (Bisgaard et al. 2023). In another study by Römges et al., 2014 the tip diameter of single solid MNs (5 to 37 µm) and its impact on the piercing process were investigated (Römgens et al. 2014). MNs with a 5 µm tip diameter seamlessly penetrated the skin, while those with larger tip diameters experienced a sudden increase in penetration depth after initial superficial insertion. A linear relationship between the force at insertion (defined as the force at a sudden decrease in measured force) and tip diameter was found.

MNs have been explored for transdermal insulin delivery, providing a less invasive option for individuals with diabetes. A study by Chen et al., 2023, investigated the use of functional insulin aspart/insulin degludec-based MNs (MNs) for the promotion of postprandial glycemic control in diabetic rats. The MNs were fabricated using a chitosan-based matrix and loaded with either insulin aspart (IAsp) or insulin degludec (IDeg). The mechanical properties, structural stability of insulin, and transdermal application characteristics of the MNs were assessed. In vivo experiments demonstrated that the MNs had comparable blood glucose control abilities to subcutaneous insulin injections. Additionally, the therapeutic properties of the MNs were investigated under various dietary conditions and application strategies. The findings suggest that the MNs have the potential to improve glycemic control, reduce hypoglycemia risk, and increase patient convenience, Fig. 4B (Chen et al. 2023).

Another study by Kim et al. 2014 investigated the development and efficacy of DMN Patches (DMNPs) loaded with Retinyl Retinoate (RR) and Ascorbic acid (AA) for wrinkle improvement. The DMNPs were fabricated using a biocompatible PVA matrix and characterized for their morphology, mechanical properties, and drug loading capacity. In vitro studies demonstrated the sustained release of RR and AA from the DMNPs, while in vivo studies showed that the DMNPs significantly improved wrinkle depth and reduced skin roughness compared to a control patch. The findings suggest that DMNPs offer a novel and effective approach to wrinkle reduction with enhanced patient convenience and comfort (Kim et al. 2014).

MN-based vaccination offers an efficient and painless alternative to traditional needle injections, potentially improving vaccine coverage and compliance. As suggested by Choi et al. 2023 investigating the efficacy of MN for delivering the hepatitis B vaccine in mice and rhesus macaques. The authors show that patches can effectively deliver the hepatitis B vaccine and induce protective immunity against hepatitis B infection in both animal models (Choi et al. 2023).

MNs may be employed for localized delivery of chemotherapy agents directly into tumours, minimizing systemic exposure and side effects. For example, the researchers developed a self-degradable MN patch capable of sustained anti-programmed death- 1 (aPD1) delivery in a controlled manner. The MN, composed of HA and pH-sensitive dextran nanoparticles (NPs), encapsulates aPD1 and glucose oxidase (GOx). The inclusion of GOx facilitates the conversion of blood glucose to gluconic acid, creating an acidic environment that triggers the self-dissociation of NPs, leading to the substantial release of aPD1. In experiments using a B16 F10 mouse melanoma model, a single administration of the MN patch induced robust immune responses compared to MN without a degradation trigger or intratumoral injection of free aPD1 with an equivalent dose. Additionally, the administration strategy proved adaptable to combination therapy, integrating with other immunomodulators (such as anti-CTLA- 4) to enhance antitumor efficacy, Fig. 4C (Wang et al. 2016).

These research articles demonstrate the potential of MNs to revolutionize drug delivery by providing more effective, painless, and convenient methods for administering vaccines and treatments.

### Fluid extraction, biosensing, monitoring analytes and bio-signal recording

In addition to drug delivery, MN is also finding applications in diagnostics. They can be used to collect samples from the body for analysis and testing purposes. In the context of fluid extraction, MNs offer a novel approach for obtaining biological samples, such as ISF or blood, with advantages including reduced pain, improved precision, and enhanced patient compliance. This makes them useful in medical settings for diagnosing diseases and monitoring health conditions (Ma et al. 2023).

**Fig. 5:**
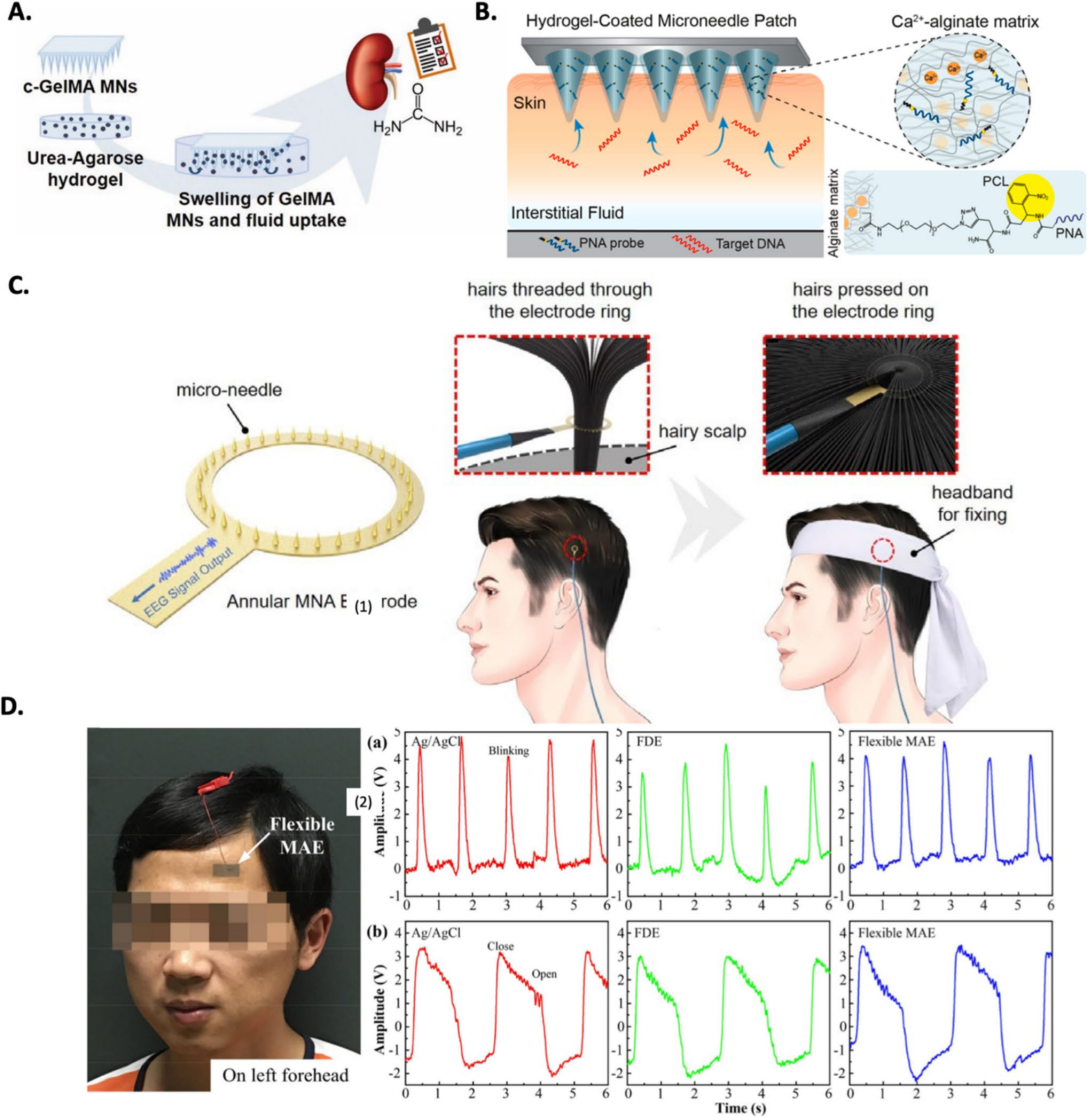
A. *In vitr*o swelling and fluid uptake by the obtained MNs using an agarose model system, adapted from Fonseca et al. (2020). **B.** Morphology of the fabricated drug-loaded MNs. MNs are functionalized with bespoke peptide nucleic acid (PNA) probes (blue) which are covalently bound to an alginate hydrogel matrix via a photocleavable linker (PCL, yellow), adapted from Ren et al. (2018). **C.** Schematic view of annular microneedles array (MNA) dry electrode and the principle of recording EEG on hairy scalp, adapted from Maia et al. 2023. **D.** EEG signals recorded by the Ag/AgCl electrode, Fixed MNA and flexible parylene-based MNA under: (**1**) the eyes blinking, and (**2**) the open and closed eyes transition, adapted from Fonseca et al. (2020)

MNs can also be used to develop biosensors. Biosensors are devices that can detect and measure biological molecules, such as proteins, DNA, and hormones. MNs can be used to immobilize biological molecules on their surface, which allows them to interact with the fluid that is extracted from the skin/cell. Current MN biosensors employ diverse detection methods and can sense a wide range of analytes, including glucose, proteins, ions, drugs, metabolites, biopotentials, and plant DNA, mainly focusing on small molecules. These biosensors have undergone rapid development, enabling fast biofluid transfer, analyte diffusion, and biorecognition within seconds or minutes (Liu et al. 2020). For example, Fonseca et al., 2020 demonstrate that gelatin methacrylate with nanofibrillated cellulose (c-GelMA) MNs can be successfully prepared by photo-cross-linking and micromolding and that they exhibit good mechanical properties and can penetrate up to 237 µm depth into the skin. In addition, the MNs can efficiently recover urea from ISF. The authors conclude that c-GelMA MNs are promising devices for sampling ISF and offline analysis of urea for point-of-care healthcare monitoring of renal complaints, Fig. 5A. Another example is described by Sulaiman et al., 2019, a method for collecting and analyzing nucleic acid biomarkers from ISF using MN arrays coated with an alginate-peptide nucleic acid (PNA) hybrid material. This minimally invasive approach offers rapid sampling, efficient biomarker capture, and versatile detection options, either on the patch itself or in solution after light-induced release, Fig. 5B (Sulaiman et al. 2019).

MNs can also be used to record bio-signals, such as electrical signals from the heart and brain. MN electrodes can be embedded in MN arrays to record bio-signals from the skin. In the field of bio-signal recording, MN electronically acting as dry electrodes has been referenced as a turnover, softening up the bottlenecks of Ag/AgCl electrodes on human pathology and physiology, such as skin preparation, skin irritation and gel drying over time (Trzebinski et al. 2012). This cutting-edge method doesn’t rely on electrolytic gel, allowing for the continuous recording of biological signals while minimizing skin trauma (Lu et al. 2022). Up to now, MN-based devices have been successfully used in Electrocardiography (ECG) and Electroencephalography (EEG) recording (Fig. 5C and Fig. 5D), allowing for real-time monitoring of vital parameters such as heart rate, glucose levels, or even brain activity (Yu et al. 2009; Kashaninejad, et al. 2021; Ren, et al. 2018). To enhance the application feasibility, the typical Si electrodes have been coated with other polymers (e.g. Polyimide) or even just metal materials (e.g. silver/silver chloride), lower impedance providers enabling less noise and larger current flow from the source to the electrodes (Li et al. 2021; Wang et al. 2017). The MNs can reduce the impedance between the skin and the electrodes, improving signal quality and minimizing skin irritation.

### MNs into microfluidic devices

**Fig. 6:**
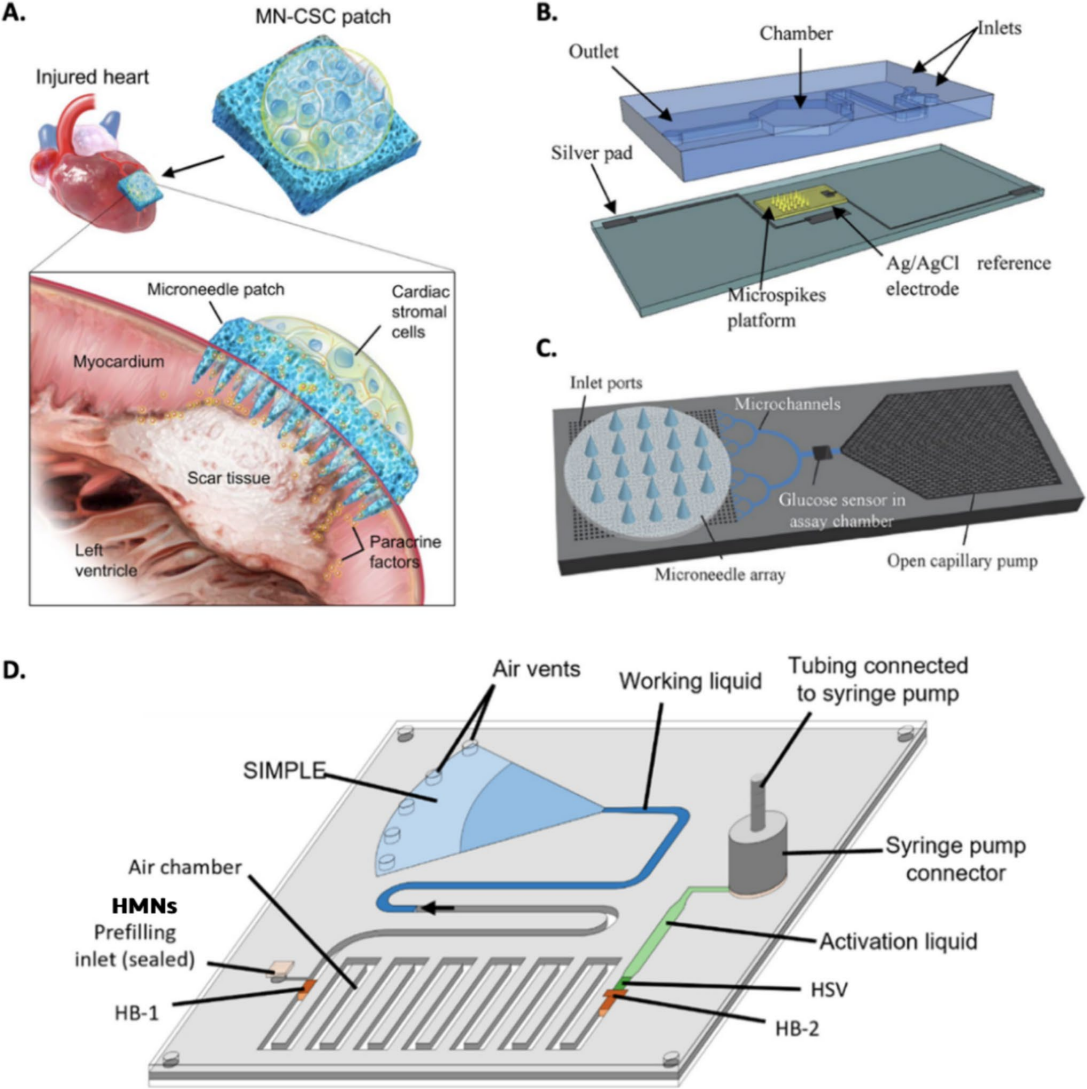
A. Schematic showing the overall design used to test the therapeutic benefits of MN-CSCs on infarcted heart adapted from Tang et al. 2018b. **B.** A schematic representation of a microfluidics-based system to study mass transport phenomena in biosensors adapted from Trzebinski et al. (2012). **C.** Schematic of the proposed fluidic system combining a porous MN array and a microfluidic chip for minimally invasive and continuous ISF sampling adapted from Takeuchi et al. (2022). **D**. Chip design of a working chip adapted from Hileghem et al. (2023)

MNs have also become indispensable tools in cell culture and tissue engineering applications. Their ability to precisely control cell morphology, enhance cell adhesion, and induce cell migration makes them ideal for manipulating and guiding cellular processes (Maia et al. 2023, 2024). It demonstrates the potential of MNs to create cell-laden microarrays, enrich and culture stem cells, and promote tissue formation and regeneration. For example, Tang et al., 2018 describe a novel MN patch for treating myocardial infarction (MI), a condition caused by heart muscle death due to lack of blood flow. The MN patch is integrated with cardiac stromal cells (CSCs), which can differentiate into cardiomyocytes, the main cells of the heart muscle. The patch is applied directly to the heart and is designed to release CSCs into the damaged area, where they can help to repair the scar tissue and restore the heart's function. The paper reports that the MN patch was able to significantly improve the functional recovery of mice with MI, Fig. 6A (Tang et al. 2018b).

Recent efforts have concentrated on MN-based devices, enabling direct sensing at the interface between the ISF and MN. In this context, electrode materials are packed into the lumen of HMN, and the electrode transducer is directly utilized at the ISF-MN interface. Integrated MN-electrode devices incorporating various transducing elements such as carbon fibres, modified carbon pastes, and platinum-coated polymer MN have been successfully employed to selectively detect a range of analytes including glucose, glutamate, lactate, hydrogen peroxide, and AA (Mohan et al. 2017; Miller et al. 2012; Windmiller et al. 2011a; Windmiller et al. 2011b). Other examples of solid Pt and Ag wires integrated with pyramid-shaped HMN have been reported.

In the scope of biomarker capture applications, MN systems commonly are composed of identification elements or probes for the selection and capture of analytes (e.g. antibodies and aptamers), thus enabling on-site analysis (Yi et al. 2021; Ni et al. 2021). The recognition mechanism and element of MN for biomarker capture are mainly immunoaffinity and an antibody, oligonucleotide, or other molecular motif-protein bond, respectively, resulting in an effective target analyte split separated from the complex biological matrix (Kang et al. 2020). To effectively capture specific biomarkers within the body, MN must possess excellent protein-antibody binding capabilities, robust mechanical strength, and biocompatibility (Wang et al. 2021a). Studies by Takeuchi et al. (2022), Trzebinski et al., 2012 and Hileghem et al. (2023), have demonstrated the feasibility of using MN-based biosensors for detecting a range of biomarkers (Fig. 6B to Fig. 6D) (Trzebinski et al. 2012; Takeuchi et al. 2022; Hileghem et al. 2023). Trzebinski et al., (2012) developed a microfluidic device that can be used to investigate factors such as the effect of different enzyme concentrations and substrate concentrations on the performance of biosensors. The device consists of a poly(dimethylsiloxane) (PDMS) microfluidic chip with microspikes embedded in it. It was used to study the performance of biosensors for the detection of glucose and lactate. They found that the microfluidic device could improve the sensitivity and selectivity of the biosensors. They also found that the microspikes could increase the stability of the biosensors, Fig. 6B (Trzebinski et al. 2012).

Takeuchi et al., (2022) designed a microfluidic chip with a capillary pump integrated into it. The chip was then connected to a porous MN array that could puncture the skin and collect ISF. The ISF was then transported through microchannels to a detection zone, where specific molecules or analytes could be analyzed. The microfluidic chip was successfully able to collect ISF efficiently and with minimal disturbance to the tissue. The chip also performed well in vitro, demonstrating its ability to monitor glucose and lactate concentrations in ISF, Fig. 6C (Takeuchi et al. 2022).

Hileghem et al., (2023) describe a novel method for fabricating HMNs that can be used for blood sampling. The HMNs are fabricated using a combination of micromilling and laser drilling techniques. The hollow design of the needles allows for the flow of blood through the needles, which can then be collected for analysis. In this specific work, the HMNs are integrated with a self-powered imbibing microfluidic pump by liquid encapsulation (SIMPLE), Fig. 6D (Hileghem et al. 2023).

These advancements hold immense promise for personalized medicine and early disease detection. Table 2 provides a simplified summary of the information discussed above.

**Table 2:** Summary of the MN material, fabrication and applications

MN type	MN material	Fabrication technology	Application
**Solid (including PMN)**	Si	Si dry-etching process Isotropic etching Anisotropic wet etching Dicing a Si substrate and then acid etching 3D laser-ablation	Transdermal drug delivery Cosmetic and dermatological treatments Microfluidics and LoC devices (PMN) Mostly used for pre-treating the skin
	Metal (e.g., stainless steel)	Laser cutting Wet etching Micromachining Metal electroplating methods	
	Polymer (e.g., PC, PDMS, PEG, Polysulfide (PSF), Polydopamine (PDA), …)	Photolithography Phase inversion Mold casting and salt leaching	
	Ceramic (e.g. Alumina (Al_2_O_3_) and Zirconia (ZrO_2_))	Micro molding and sintering lithography	
	Others (Glass, Cellulose acetate, …)	Glass pulling. Phase inversion	
**Coated**	Immersing or applying a high-viscosity aqueous solution that includes a surfactant, the active agent, and a stabilizing agent onto the MN Dipping the MN once or multiple times into a coating solution. Alternatively, each individual MN can be immersed in a microwell that contains the drug solution. Another method involves applying a pre-formed film of the drug solution onto the MN using a roller	Delivery of multiple agents through the same formulation Wearable devices for monitoring and biosensing	
**Dissolvable**	Photolithography Deep X-ray lithography Drawing lithography Micromolding and melt casting Droplet born air blowing Two-photon polymerization 3D printing	Application involves only a single step Cosmetic and dermatological treatments Glucose monitoring Gene and protein delivery Long-term therapy with improved patient compliance	
**Hydrogel/Swelling**	Photolithography Deep X-ray lithography Dry etching Wet etching Metal electroplating Drawing lithography Two-photon polymerization Micromolding	Drug delivery Biomedical sensing Transdermal vaccination Personalized medicine Cosmetic and dermatological treatments	
**Hollow**	MEMS techniques-laser micromachining Deep reactive ion etching of Si Integrated lithographic molding technique Deep X-ray photolithography Wet chemical etching and micro-fabrication	Drug delivery Analysis of large-sized molecules like proteins, vaccines, oligonucleotides Cosmetic and dermatological treatments Wearable sensors Microfluidic devices	

## Conclusions

MN technology has become an innovative and adaptable instrument in the biomedical field, providing novel solutions for various challenges in drug delivery, diagnostics, and therapeutic applications. These innovations in MN have led to advancements in the materials used and their applications, including dissolving and hydrogel-forming needles. Each type of MN provides unique benefits and applications tailored to the specific requirements of the study or treatment (Meng et al. 2020b). If the goal is systemic drug delivery, MNs made from polymers or metals might be suitable due to their robustness and ability to deliver precise doses. For vaccination purposes, biodegradable polymer MNs are often preferred because they enhance the immune response and offer safety advantages. In cosmetic applications, such as anti-aging or acne treatments, biocompatible materials like hydrogels or certain polymers are often the materials of choice. For biosensing applications, silicon or metal MNs are typically used due to their precision and conductivity.

Applications include delivering skincare products in cosmetics, transdermal insulin delivery, offering an efficient and painless alternative to traditional needle injections for vaccinations, and directly delivering chemotherapy agents into tumors. MNs can also be integrated into biosensors and microfluidic platforms, such as organ-on-a-chip systems, enhancing advancements in personalized medicine. MNs exhibit substantial potential for growth and development due to their versatile manufacturing techniques. These techniques range from traditional methods such as photolithography to advanced methods including 3D printing and hydrogel formation with drug encapsulation. This versatility enables the customization of MN structures tailored to specific objectives, functions, or applications.

Regarding the transition from experimental studies to clinical implementation, several key clinical parameters must be carefully addressed.

The MN must demonstrate high reproducibility and precision, ensuring consistent drug delivery across different patients and treatment conditions. Biocompatibility and safety play another important role when selecting the materials to be used in MN fabrication, particularly those with responsive properties, which must undergo rigorous testing for cytotoxicity, immunogenicity, and long-term stability in the human body. Additionally, current MN-based detection systems primarily offer qualitative results, which limits their clinical utility. Future developments should prioritize quantitative biomarker monitoring aligned with validated diagnostic thresholds, enabling accurate and comparable measurements across clinical settings.

Furthermore, large-scale production and cost-efficiency must be addressed to ensure widespread accessibility. Without this, even the most technologically advanced systems may face barriers to adoption.

## Current challenges and future directions

MNs has emerged as a promising technology for drug delivery and medical diagnostics. However, their research and clinical applications face several significant limitations. Firstly, there is a need for extensive clinical testing to ensure the safety and efficacy of MN-based products, which can be a time-consuming and costly process. Additionally, the manufacturing of MNs at a large scale remains challenging, impacting their cost-effectiveness and widespread accessibility. Furthermore, regulatory hurdles and intellectual property issues can hinder market entry and growth (Waghule et al. 2019; Ita 2015).

Current MN-based sampling and detection methods primarily offer qualitative analysis based on parameters such as fluorescence intensity, absorbance, or normalized quantities. This limitation in providing quantitative data hinders the comparison of biomarker concentrations across experiments and labs, constraining biomedical research and the establishment of standardized clinical biomarker cut-off values. Detecting protein biomarkers in ISF is challenging due to the dense tissue environment, affecting analyte antibody binding kinetics and slowing target biomolecule diffusion to the MN surface. Consequently, this reduces the likelihood of capturing analytes and lowers signal intensity (Zhang et al. 2019; Wang et al. 2021b). A knowledge gap exists in this domain, impeding the clinical implementation of MN technology. MN-based assays often suffer from limited sensitivity, low detection limits, and potential cross-reactivity with other ISF molecules (Chen et al. 2023). Improving guidelines for MN-based products is necessary. Future MN monitoring devices should prioritize easy fabrication and portability for medical sensing applications, emphasizing long-term sensor accuracy and linking concentration changes with physiological significance (Horowitz et al. 2020). By leveraging the unique properties of MN, researchers can gain a deeper understanding of the body’s state and make informed decisions in healthcare and medical research (Zhang et al. 2021; Kim et al. 2019; Najmi et al. 2021; Invernale et al. 2014;Ribet et al. 2018).

Current MN-based drug delivery platforms mainly rely on passive diffusion or controlled material degradation to release therapeutic agents. However, these approaches lack precise control over release timing, dosage, and depth of drug penetration (Amani et al. 2021). The integration of external stimuli, such as light, ultrasound, magnetic fields, and gas bubbles, presents an opportunity to enhance drug delivery efficiency and achieve on-demand, localized treatment (McGrath et al. 2011). These technologies enable remote actuation, targeted release, and real-time adaptability, improving the overall effectiveness of MN-based therapies. Before these sensors can be clinically applied, they must undergo stringent cytotoxicity tests to guarantee their safety. To enhance their effectiveness, the materials used in MN sensors should possess the ability to minimize immune system rejection and resist microbial infections (Woedtke et al. 2002; Zhu et al. 2020).

The accuracy of detecting biomarkers depends on the specificity and sensitivity of the detection method used, such as electrochemical sensors. However, these sensors may experience reduced sensitivity over time due to fluid flow washing away the recognition molecule or cleaning steps between readings. Additionally, the devices’ fabrication processes are time-consuming and involve multiple steps and additional sealing layers to enhance their overall thickness (Mohan et al. 2017). These limitations need to be considered for this application. Despite this, the research performed in this field have been demonstrated the potential and versatility of microfluidic devices for biomarker detection.

The integration of MNs within microfluidic devices will be crucial for advancing MN technology in healthcare. However, some challenges such as clogging, biocompatibility, fluid leakage, multiple fabrication steps, and high costs could be an obstacle in rapid advancements. These complexities can adversely affect the reliability and repeatability of manufacturing such devices. Ensuring precise alignment of MN with fluidic channels is vital to ensure smooth fluid flow and proper functionality. Hence, it is essential to select suitable materials for MNs that align with the practical requirements of the intended application. Additionally, more clinical trial data may be required to provide the pharmaceutical industry with a level of confidence that will stimulate further investment in the development of MN products.

## Data Availability

No datasets were generated or analysed during the current study.

## Abbreviations

aPDI: Anti-programmed death-1
AA: Ascorbic Acid
CMSs: Coated Microneedles
CSCs: Cardiac Stromal Cells
c-GelMA: Gelatin methacrylate with nanofibrillated cellulose
DLP: Digital Light Processing
DMNPs: Dissolving Microneedles Patches
DMNs: Dissolving Microneedles
DNA: Deoxyribonucleic Acid
DRIE: Deep Reactive Ion Etching
EEG: ElectroEncephaloGram
FDM: Fused Deposition Modelling
GOx: Glucose oxidase
HA: Hyaluronic Acid
HfMNs: Hydrogel-forming Microneedles
HMNs: Hollow Microneedles
ISF: Interstitial Fluid
MNs: Microneedles
MNA: Microneedles Array
PNA: Peptide Nucleic Acid
NPs: Nanoparticles
PC: Poly(carbonate)
PDMS: Poly(dimethylsiloxane)
PLA: Poly(lactic acid)
PMMA: Poly(methyl methacrylate)
PNA: Peptide Nucleic Acid
PMNs: Porous Microneedles
PP: Poly(propylene)
PS: Poly(styrene)
PVA: Poly(vinyl alcohol)
PVa: Poly(vinyl acetate)
PVP: Poly(vinyl pyrrolidone)
RNA: Ribonucleic acid
RR: Retinyl Retinoate
Si: Silicon
SLA: Stereolithography
SMNs: Solid Microneedles
